# Genetic test feedback with weight control advice: study protocol for a randomized controlled trial

**DOI:** 10.1186/1745-6215-13-235

**Published:** 2012-12-06

**Authors:** Susanne F Meisel, Rebecca J Beeken, Cornelia HM van Jaarsveld, Jane Wardle

**Affiliations:** 1 Health Behavior Research Centre, University College London, 1-19 Torrington Place, London, WC1E 6BT, UK

**Keywords:** Genetic test feedback, Obesity prevention, DNA test, Behavior change

## Abstract

**Background:**

Genetic testing for risk of weight gain is already available over the internet despite uncertain benefits and concerns about adverse emotional or behavioral effects. Few studies have assessed the effect of adding genetic test feedback to weight control advice, even though one of the proposed applications of genetic testing is to stimulate preventive action. This study will investigate the motivational effect of adding genetic test feedback to simple weight control advice in a situation where weight gain is relatively common.

**Methods/design:**

First-year university students (n = 800) will be randomized to receive either 1) their personal genetic test result for a gene *(FTO)* related to weight gain susceptibility in addition to a leaflet with simple weight control advice (‘Feedback + Advice’ group, FA), or 2) only the leaflet containing simple weight control advice (‘Advice Only’ group, AO).

Motivation to avoid weight gain and active use of weight control strategies will be assessed one month after receipt of the leaflet with or without genetic test feedback. Weight and body fat will be measured at baseline and eight months follow-up. We will also assess short-term psychological reactions to the genetic test result. In addition, we will explore interactions between feedback condition and gene test status.

**Discussion:**

We hope to provide a first indication of the clinical utility of weight-related genetic test feedback in the prevention context.

**Trial registration:**

Current controlled trials ISRCTN91178663

## Background

‘Personalized’ healthcare aims to offer specific, targeted advice for disease prevention based on genetic risk status, [1,2]. Addition of information about genetic risk markers to standard care has been put forward as a way of empowering individuals to take responsibility for health maintenance, thereby maintaining quality of life and lowering healthcare costs [3]. To date, the clinical utility of most genetic risk markers is assumed to be low because of the small effect sizes of genes identified for ‘common’ conditions. However, genetic testing can already be purchased over the internet without involvement of healthcare providers (for example, http://www.23andme.com; http://www.deCODEme.com), suggesting that consumers are interested in this kind of information. The idea that genetic test feedback could promote behavior change is consistent with psychological theories (such as protection motivation theory [4]) that identify perceptions of risk as important for motivating risk-reducing actions. Within this model, however, a lower-risk genetic test result might reduce motivation. An alternative perspective is that ‘genetic determinism’ (the idea that genetic effects cannot be modified) could lead to a fatalistic attitude towards health maintenance for example, [5] in the case of a higher-risk result, whereas a lower-risk result could induce a false sense of immunity and complacency for example, [6]. Furthermore, there are concerns that individuals may discount their genetic result if it does not match their preconceived ideas about illness development [7]. To date, few studies have investigated the consequences of genetic test feedback for common conditions. One approach uses ‘vignettes’ in which people imagine receiving a genetic test result and anticipate their reactions. Results for smoking cessation and prevention of weight gain suggest that higher-risk feedback increases motivation to change behavior and does not appear to induce fatalism [8-10], while lower-risk feedback does not appear to induce complacency, and may even have a mildly motivating effect [9]. However, the gap between expressed intentions in the vignette context and actual behavior may be wide, so at best, these results only give an indication of the effects of actual test feedback.

‘Real’ genetic test feedback has received most attention in the smoking cessation field following the early discovery of genes coding for enzymes that reduce risk for lung cancer (*GSTM1* and *CYP2D6*). There has been evidence of increased motivation to quit following genetic test feedback [11,12], although a recent Cochrane review of five clinical studies failed to show any statistically significant effects on quitting when incorporating genetic feedback results into smoking cessation programs, either in the short term (two weeks), or in the longer term (six months) although study heterogeneity limited the interpretation of the results [13]. However, the physiological and psychological drivers of nicotine addiction make smoking cessation particularly difficult to achieve, and quit attempts are marked by frequent relapse [14]. Adding genetic test feedback in this context might be less influential than for other health promoting behaviors. The positive feature of these results was that there was no evidence that smokers receiving lower-risk results thought of themselves as immune to lung cancer for example, [12,15]. This corresponds with findings from vignette studies, and indicates that complacency may not be a major concern. There is some evidence that inclusion of genetic test feedback for risk of obesity into clinical care may be beneficial. Feedback on a gene implicated in weight gain (*bA3R*) resulted in increased motivation to lose weight in a small sample of obese individuals [16]. Another small qualitative study giving feedback on the *FTO* gene (also linked with weight gain), found that feedback increased motivation to lose weight (SFM and JW, unpublished). This study also found that the ‘scientific’ nature of the gene test result helped overweight participants feel less sense of personal failure and more confidence in managing their ‘condition’. A similar finding was reported in a community sample of overweight and obese adults, where genetic feedback reduced guilt and self-blame [17]. Raising awareness of the genetic etiology of obesity also helps minimize negative stereotyping by healthcare professionals [18].

Thus far, most research has centered on the reactions of already affected individuals, but a key proposed application for genetic feedback is *prevention* of ill health. The one study in this area analyzed responses to receiving results from a direct-to-consumer genetic test for a panel of diseases in over 2000 people who volunteered to be followed up in return for purchasing their test at a reduced rate [19]. Neither higher nor lower genetic risk for the panel of diseases was associated with changes in anxiety or health behaviors, nor were there changes in the number of health screening tests although intended use of health screening increased in the whole group. However, the participants in the study are likely to have been ‘early adopters’ of genetic testing who may be more health conscious than average and have less scope to change, supported by the fact that most of them were already following a low-fat diet. It is also possible that receiving results for a whole panel of diseases has a different effect from receiving results for a single condition because, inevitably, any high risk results are likely to be balanced by lower risk for others diseases, which may generate an overall null effect on risk perception. The present study will therefore investigate the impact of giving genetic risk feedback for a single condition, overweight, in conjunction with weight control advice, in a healthy, young adult sample entering a life stage where risk of weight gain is raised, to discover whether genetic feedback provides a ‘nudge’ toward unhealthy weight gain prevention. We will test the effect of adding feedback for the *FTO* gene, which has modest effects on weight gain and risk of obesity [20], to simple weight control advice. We use the university context because of evidence that first-year students have a high risk of weight gain (sometimes termed the ‘Freshman 15’ or ‘Freshman 5’) and low intentions to implement healthy behaviors [21,22].

### Study objectives and hypotheses

#### Primary research objective

The primary aim of the study is to test the hypothesis that adding genetic test feedback to weight gain prevention advice will result in higher motivation to prevent weight gain one month after receiving test feedback compared with receiving weight gain prevention advice alone.

#### Secondary research objectives

The secondary objectives are to assess differences in adherence to the advice and weight change from baseline to eight month follow-up in those receiving genetic test feedback and weight gain prevention advice compared with advice alone. We hypothesize that participants receiving genetic test feedback, regardless of risk status, will be more adherent to the advice and gain less weight over the study period than those receiving weight gain prevention advice alone. Within the group of participants receiving feedback and advice, we will also explore psychological reactions immediately after receiving genetic test feedback, and interactions between weight status and gene test status. We hypothesize that those receiving a higher-risk genetic test result will show higher negative affect but not higher fatalism in response to their genetic test result compared to those receiving an ‘average-risk’ genetic test result. We also predict that overweight participants receiving a ‘higher-risk’ result will value having an explanation for their weight more than normal weight participants, and that overweight participants will be more motivated to prevent further weight gain in response to a higher-risk genetic test result than those receiving an ‘average-risk’ result. All secondary objectives are exploratory, because of limited power.

## Methods/design

### Study design

**Figure 1:**
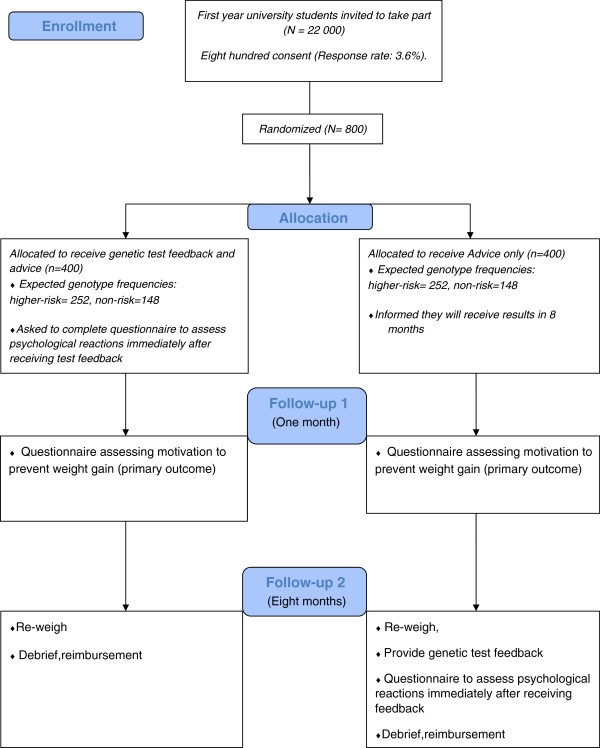
Flowchart of study procedures.

The design will be an open, two-arm, individually-randomized, controlled trial comparing the effects of genetic test feedback for risk of weight gain combined with weight control advice (FA) with a control condition of giving weight control advice only (AO). The control group will receive their genetic test result at the end of the study. A summary of the study procedures is shown in Figure 1. Participants consenting to take part in the study will be randomized and offered the following:

#### Intervention group (‘Feedback and Advice’ group)

Following baseline measurements, participants randomized to the ‘Feedback and Advice’ group will receive their gene test result in an email, as used by internet-based genetic testing services. This format was used in a previous study (SFM and JW, unpublished) and found acceptable to participants. The result letter will be sent as an email attachment so that participants can open and read it at a convenient time. The letter will contain the personal result in addition to information about prevalence in the population. The email will also include a short information leaflet written in simple language, giving a brief overview about the *FTO* gene, its mode of inheritance, and magnitude of influence on body weight. Contact details of the research team will be included for questions about the test results. The *FTO* information leaflet and the result letter were piloted in a general population sample that included both men and women of various ages and ethnicities (n = 35), and has been used in previous work (SFM and JW, unpublished). Both were deemed informative, comprehensive and easy to understand. Readability was assessed with the Flesch Reading Ease formula and the Flesch-Kincaid Grade Level formula, and letter and leaflet received a score of 71.0 and 67.0 respectively, which translates to the reading level expected in grade 6 (age 12). Participants will also receive weight control advice in the form of a short leaflet containing practical ‘tips’ on how to avoid weight change when starting university. The format and design is based on habit formation model [23], and is currently being evaluated in a large population-based sample [24]. Readability of the leaflet was tested with the Flesch Reading Ease formula and the Flesch-Kincaid Grade Level formula, and found to be high (71.1), comparable to the reading level expected in grade 6 (age 12).

#### Control group (‘Advice Only’ group)

Following baseline measurements, participants randomized to the ‘Advice Only’ control group will receive an email at the same time as the FA group informing them that they will receive their test result by the end of the academic year, resembling a ‘wait-list control’ group for the gene test feedback condition. The weight control advice leaflet will be attached to the email, matching the format of the intervention group. Ethical concerns about withholding weight control advice from individuals at increased risk of weight gain, and the likelihood of contamination between groups, led to the decision to send the leaflet to all participants and therefore not to have a ‘no treatment’ control group that receives neither leaflet nor genetic feedback. The gene test result will be sent out in identical format to the intervention group at the end of the academic year after collection of follow-up anthropometric data is complete.

### Randomization

All consenting participants will be randomized to ‘FA’ (intervention) or ‘AO’ (control) after baseline data collection is complete and records have been anonymized using serial numbers. For group allocation, we will use the randomization function in the Statistical Package for Social Sciences (SPSS 19 for Windows®; SPSS Inc., Chicago, IL, USA), which randomly assigns a set percentage of cases to a specified number of groups. Each group will consist of 400 participants, and based on population frequencies of *FTO* we expect 252 to have at least one higher-risk (A) allele and 148 to have the lower-risk (TT) genotype. Randomization will take place after data collection of each wave is complete, but before the DNA analyses are conducted. Experimenter bias is unlikely, as records are anonymous and not easily identifiable. Although neither researchers nor participants will be blind to group allocation, follow-up weights will be measured objectively and print-out records kept, which excludes inadvertent manipulation of records. Questionnaire data will be collected online, which minimizes the risk of missing data or inadvertent manipulation.

### Minimizing other sources of bias

Participants who will select themselves into the study are likely to be more positive towards genetic testing than the average student population. However, if genetic testing were offered through a healthcare provider or over the internet, the same situation would hold; that is, only interested individuals would choose to take part, and this situation therefore should not unduly influence the generalizability of the results.

### Recruitment and consent

Ethical approval was granted by the University College London Research Ethics Committee for non-NHS research in September 2010. Participants will be recruited at the beginning of two consecutive academic years. Potential participants will be recruited through emails sent to all first year students (approximately 22,000) enrolled at University College London (UCL) inviting them to take part in a study where they will receive information on their personal genetic risk for weight gain susceptibility. A ‘collection station’ will be set up at UCL in the first two weeks of term where participants can receive detailed information about the study, check eligibility, enroll, and give saliva for DNA analysis. To ensure that participants are aware of the details of the study, information contained in the information sheet will be reiterated by a researcher. Participants will be told that the DNA analysis is only for one gene (*FTO*) which is related to a small increase in weight gain susceptibility and that students will be randomly allocated to receive their results either during term 2 (intervention group) or at the end of the academic year (control group). Participants will be informed that their samples will be destroyed on completion of the study and not stored for further analyses. Written consent will be obtained before participants give saliva for DNA analysis. The right to withdraw from the study without giving reason will be respected at all times, and saliva samples will be destroyed immediately after participant withdrawal.

### Participants

Participants will be a volunteer sample (n = 800) of first year university students.

#### Inclusion and exclusion criteria

We will include all interested first-year students between 18 and 25 years who are able to give informed consent. We chose this age range because the association between *FTO* and BMI has been found to peak in early adulthood, with less effect thereafter [25]. As *FTO* has been found to have effects in all major ethnic groups, we will not exclude participants based on ethnicity. We anticipate that a small number of individuals suffering from eating disorders may express interest in taking part, because eating disorders are relatively common in this age group [26]. However, participants have the option of declining to see weight and body composition results. If they opt to receive their body composition assessment, they will get brief, personal feedback with it. We will be careful to avoid discriminating against anyone based on their weight (high or low) because this may impair help seeking. Instead, we will offer brief, general advice on available resources to all participants, so that those who wish to find help can do so. If a participant is distressed by the result, the principal investigator, who is a clinical psychologist, will be available for intervention. Results from earlier qualitative work indicate that gene test feedback may be helpful even for individuals with a history of eating disorders, and that provision of information should be sufficient to eliminate the risk of exaggerating tendencies for disordered eating (SFM and JW, unpublished).

### Proposed study duration

The study will run over three university terms (eight months).

## Measures

### Baseline

#### Body composition

Participants will be asked to remove shoes, socks and heavy items, but stay otherwise fully clothed. Height will be measured, rounded up to the nearest centimeter, using the Leicester Height Measure, a standardized instrument for determining height. Weight and body fat will be assessed using the TANITA TBF-300 MA Body Composition Analyzer (Tanita, Tokyo, Japan). This uses electrical impedance to assess body fat which compares well to other measures of body composition such as whole body magnetic resonance imaging and dual X-ray absorptiometry [27]. Participants can opt to receive a printout of the results together with an explanation by the research team or not.

#### DNA Collection and genotyping

Participants will be asked to give a saliva sample for DNA collection by placing some sugar onto their tongue to stimulate saliva flow and then ‘drooling’ into a plastic tube to generate 1.5-2 ml of saliva. Samples will be coded with a unique identifier number so that they are anonymous but can be linked back to questionnaire data. DNA will be isolated and extracted from saliva. Genotyping of *FTO rs9939609* will be performed using TaqMan; details of extraction and analysis methods have been published elsewhere [28]. Samples will be analyzed at the Institute of Metabolic Sciences, Cambridge, which is a certified research laboratory.

#### Baseline questionnaire

The baseline questionnaire will be sent by email after anthropometric data and saliva have been collected. It will include demographic and health-related measures (age, gender, course of study, year of study, family history of overweight), and standardized questionnaires assessing the following: *Diet quality* will be determined using the using the DINE, which is a self-report food frequency questionnaire, commonly used in clinical practice, assessing dietary fat, fiber and fruit and vegetable content [29]. *Eating behavior* will be assessed with the Dutch Eating Behavior Questionnaire (DEBQ), which determines tendencies for emotional eating, restraint eating and external eating [30]. *Physical activity* will be assessed with the Recent Physical Activity Questionnaire (RPAQ), which assesses time and frequency for a range of physical activities for commute and leisure [31]. *Stress* will be determined using the Perceived Stress Scale-4 (PSS-4, [32]. The PSS-4 is a short version of the 20-item perceived stress scale, with good psychometric properties [33]. Four items will assess *worry about weight gain*[34] and *diet self-efficacy*[35]. All questionnaires have sound psychometric properties and are commonly used in clinical practice.

### Follow-up

#### Primary outcome

The primary outcome is motivation to engage with weight gain prevention. It will be assessed using a validated measure of readiness for behavior change [36,37] adapted to relate to prevention of weight gain. Individuals will be asked: *‘*Please mark with an ‘X’ the statement out of the next four that best describes you’: ‘I am not trying to control my weight, and I have no intention of doing so in the next month’; ‘I am not trying to control my weight, but I am thinking of doing something in the next month’; ‘I started to try to control my weight within the last month’; or ‘I have been trying to control my weight for more than a month’. Responses will be used to classify individuals into one of the five stages of behavior change outlined by Prochaska and DiClemente [38]. Participants will also be asked to rate their agreement with one statement each relating to motivation to prevent weight gain, perceived ease of weight control, and the priority they are giving to weight control (ranging from ‘never’ to ‘always’*)*. The primary outcome will be measured one month after the intervention group receives their genetic test result. We chose the respective time frame, because we saw it as crucial that participants would have had sufficient time to think about their result, to ask any questions, and to implement eventual behavior change. We will exclude participants who controlled their weight for more than one month, because the intervention will not be applicable to them.

#### Secondary outcomes

Secondary outcomes are psychological reactions to genetic test feedback and actual behavior change as assessed by adherence to the individual weight control tips in the leaflet. Immediately after receiving genetic feedback (FA group after allocation, AO group after 8 months, see Figure 1), participants will complete a questionnaire concerning their psychological reactions using 18 items adapted from the vignette study by Meisel *et al*. [9]. One example is ‘Knowing my FTO gene test result provides me with an explanation for my body weight’ (strongly disagree to strongly agree).

#### Adherence to the weight loss tips

Adherence to the weight loss tips will be assessed by asking: ‘How often in the last month did you… (watch portion sizes, avoid second helpings, slow down your eating, avoid eating mindlessly/focus on your food, pass up extra snacks between meals, avoid sweet drinks or chose a lite drink, integrated some physical activity into your day)’*.* Response options were given on a Likert scale, ranging from ‘never’ to ‘always’*.*

#### Body composition

Follow-up anthropometric measurements will be collected at eight-month follow-up as at baseline.

#### Comprehension of the genetic test result

Immediately after receiving their genetic test feedback, participants will be asked what their genetic test result was (AA, AT, TT, I don’t know), and whether their test result puts them ‘at lower/average/higher risk for gaining weight’, also including the option ‘don’t remember’ to assess comprehension of the test result.

### Minimizing drop out

We anticipate about half the participants not returning for follow-up measures because attrition rates of this magnitude have been reported from other longitudinal studies with students for example, [39,40]. To minimize attrition, we will send several email reminders; this has been shown to increase follow-up rates [41]. We will also offer a modest incentive (£5 voucher) to everyone who fills out the follow-up questionnaire *and* returns for the final weighing session. We feel that this is justified by the time and effort participants spend taking part in the study.

### Sample size and assumptions

A power calculation conducted *a priori* using *GPower* (version 3.1; http://www.psycho.uni-duesseldorf.de/abteilungen/aap/gpower3/download-and-register)showed that a total sample size of n = 340 should suffice to detect a small effect (η = 0.25) for motivation to prevent weight gain between ‘Feedback and Advice’ and ‘Advice Only’ group with 95% power or greater at the 5% significance level, accounting for an attrition rate of 60% (based on research with other student samples). We chose the small effect size based on data from the vignette study [9] and on *FTO*s moderate effects on weight.

### Statistical analyses

Differences between completers and non-completers of follow-up will be assessed with chi-square tests for categorical variables and independent-samples *t*-tests for continuous variables. Participants who had been controlling their weight for more than one month will be excluded from analysis, because the intervention will not be applicable. Correct recall of the test result will be assessed with a chi-square test. Associations between genotype status and correct recall will be assessed with a one-way ANCOVA, including age, gender and body mass index (BMI) as covariates, as recall may affect subsequent reactions.

#### Primary

Ordinal logistic regression will be used to assess the difference in ‘Feedback and Advice’ *versus* ‘Advice Only’ group for motivation to prevent weight gain. Additional predictor variables will include age, gender and weight status at baseline, because these may influence results.

#### Secondary

Results from all secondary analyses will be considered as exploratory. One-way ANOVAs, followed by one-way ANCOVAs will be used to examine immediate psychological reactions to genetic test feedback in the sample overall once the AO group participants have received their genetic test results. Repeated-measures ANOVAs will be used to determine change in anthropometric measures within groups over time in the sample overall. Anthropometric differences between groups will be determined using one-way ANCOVA. Age, gender and baseline BMI will be included as covariates. Changes in diet quality, eating behavior and physical activity in the sample overall will be examined with repeated-measures ANOVAs. Changes between groups will be examined using one-way ANCOVAs, including age, gender and baseline BMI as covariates. Group differences in actual behavior change (as measured by adherence to the individual tips) will be assessed with a one-way ANCOVA. In addition, we will build an additive score of the total number of tips adhered to at least ‘occasionally’ and compare this in intervention and control group with a one-way ANCOVA. Age, gender and baseline BMI will be included as covariates. Bonferroni corrections will be used for multiple comparisons in all analyses. Within the ‘Feedback and Advice’ group, *FTO* status will be dichotomized into high/average risk, with those having at least one risk allele being classified as ‘higher-risk’. Ordinal regression analyses will be used to examine effects of risk status on motivation to control weight, as well as interactions between risk status and baseline BMI. Age and gender will also be included as predictor variables in the model. Finally, we will assess predictors of motivation to control weight with a multinomial regression analysis. We will include all potential predictors, including age, gender, *FTO* status, as well as stress, eating behavior, baseline BMI, and weight satisfaction.

## Discussion

The disappointing results from most trials of weight gain prevention warrant development of novel methods to engage individuals with weight control. Genetic test feedback has the potential to be a tool that could help raise awareness of susceptibility to weight gain. The increasing popularity of genetic testing to ‘personalize’ medicine makes evaluation of its effects imperative. The current trial uses a rigorous study design, taking ethical and practical concerns into consideration. For example, we chose to test the impact on motivation after one month instead of immediately after receiving feedback, to give participants sufficient time to think about their test results and to ask any questions. This may provide a more accurate measure of the effects of test feedback than the possibly transient ‘spur of the moment’ reactions occurring immediately after receiving the genetic test result. However, there are a number of limitations. The study uses a young, healthy, well-educated sample, which allows for investigating the effects of feedback but limits generalizability of the results to the wider population, particularly those with lower health literacy. Control over food choices in this study is limited due to the university setting (the majority of students live in catered halls). This may affect the ease with which weight gain prevention can be achieved and results may differ in settings where control over food intake is higher. Nonetheless, results from the current sample are of interest, particularly because they provide an indication for the effect of testing for prevention of unhealthy weight gain in a population where weight gain is common. With this study, we hope to provide a first indication of the potential benefits of inclusion of genetic testing into weight management advice, laying the groundwork for future research evaluating the clinical utility of genetic testing for the prevention of ill health.

## Trial status

Recruitment of participants is in progress.

## Abbreviations

ANCOVA: Analysis of covariance; ANOVA: Analysis of variance; AO: Advice only group; BL: Baseline; BMI: Body mass index; DEBQ: Dutch Eating Behavior Questionnaire; DNA: Deoxyribonucleic acid; FA: Feedback and advice group; *FTO* gene: Fat mass and obesity associated gene; FU: Follow-up; NHS: National Health Service; PSS-4: Perceived Stress Scale-4; RPAQ: Recent Physical Activity Questionnaire; SPSS: Statistical Package for Social Sciences; UCL: University College London.

## Competing interests

All authors declare that they have no competing interests.

## Authors’ contributions

SFM and JW drafted the manuscript. All authors designed the study and revised the manuscript. RJB provided advice on trial design. CHMvJ provided statistical advice. SFM is responsible for data collection and statistical analysis. JW is the principal investigator and grant holder. All authors read and approved the final manuscript.
